# The History of the Behavior Analyst Certification Board’s Ethics Codes

**DOI:** 10.1007/s40617-023-00803-2

**Published:** 2023-05-16

**Authors:** Tyra P. Sellers, Holly A. Seniuk, Sarah N. Lichtenberger, James E. Carr

**Affiliations:** 1 Association of Professional Behavior Analysts, Highlands Ranch, CO USA; 2 Behavior Analyst Certification Board, 7950 Shaffer Parkway, Littleton, CO 80127 USA

**Keywords:** BACB, Certification, Ethics, Ethics codes, History

## Abstract

As the primary certification organization in behavior analysis, the BACB has published nine ethics-based documents and codes since its inception in 1998. The ethics standards in these publications have served as the basis of ethics education and disciplinary enforcement in applied behavior analysis for over 2 decades. As it is important for developing professions to document their evolution for later historical analysis, the purpose of this article is to describe the development and evolution of the BACB’s ethics-based documents and codes.

From 1985 until the early 2000s, Florida operated the first successful certification program for practicing behavior analysts, the Florida Behavior Analysis Certification Program (Starin et al., 1993). By the mid-1990s, the program had been so successful that state departments and professional associations in California, Texas, Pennsylvania, New York, and Oklahoma arranged to use Florida’s examinations to replicate similar certification programs in their own states (Johnston et al., 2017). At this point, it was evident that there was a need for a national behavior analyst certification program. Indeed, “as other states became increasingly interested in using the Florida examination, Florida officials decided that it would be wise for an appropriate entity to accept national responsibility for the examination program” (Johnston & Shook, 2001, p. 78). Dr. Gerald (Jerry) Shook,^1^ one of the original architects of the Florida certification program, founded the Behavior Analyst Certification Board® (BACB®) in 1998 and contracted with Florida to eventually assume its certification program operations. By 2003, all six state-based certification programs had ceased operating their own certification programs and transferred their certificants to the BACB (Johnston et al., 2017).

The first BACB certification requirements constituted an elevation above what any of the six states that operated certification programs had required. For example, the initial Board Certified Behavior Analyst® (BCBA®) requirements included a master’s degree or higher, 180 hr of behavior-analytic coursework (approximately four 3-credit semester courses), and 12 months of supervised experience or 18 months of mentored experience (Johnston & Shook, 2001). Although BACB certificants were required to adhere to a set of disciplinary standards (see Professional [Disciplinary] Standards [1999] below), there was no formal BACB ethics code at that time.

Even though the initial BCBA certification requirements were more rigorous than any of the certification programs in the original six states, the requirements—a master’s degree, four graduate courses in behavior analysis, 12–18 months of loosely defined practical experience) were arguably lower than many in the profession would have wanted at the time. The primary rationale for a strategically lower starting point was the considerable variability in where and how behavior analysts had been trained. In general, behavior analysts received their training from a few dozen university programs, most of which were not explicitly programs in behavior analysis. This meant that the numbers and types of courses taken in behavior analysis were highly variable. In addition, there were no standards for experiential training in the profession, resulting in considerable variability in the duration and quality of behavior analysts’ practical experiences, and in how and how often they were supervised. Given the considerable variability of training experiences at the time, substantially higher requirements than the initial BCBA requirements might have prevented qualified behavior analysts from obtaining certification. Thus, the BACB’s initial requirements permitted many existing behavior analysts to become certified, after which training programs could reorganize their didactic and experiential training such that later students would more readily qualify for certification.

After the BACB’s initial certification requirements were established, the organization’s ongoing strategy was (and is) to have subject matter experts (SMEs) conduct periodic reviews of its requirements to determine if and how they should be revised (Johnston et al., 2014). This approach permitted incremental revisions that were sensitive to changes in the profession at the time of each review. Periodic SME review is the most common way certification organizations approach revisions to their requirements and is also a core standard of the National Commission for Certifying Agencies (NCCA) from which the BACB received accreditation of its BCBA and Board Certified Assistant Behavior Analyst® (BCaBA®) certification programs in 2007, and Registered Behavior Technician® (RBT®) certification program in 2016.

The BACB’s certification requirements have generally increased in a steady manner over the last 2 decades. As an example, the 2022 BCBA certification requirements under the most common pathway (Pathway 2) include a master’s degree or higher, 315 hr of graduate-level behavior-analytic coursework, and at least 1,500 hr of supervised fieldwork. In addition, BCBAs must obtain 32 hr of continuing education in their 2-year certification cycle (including ethics and supervision content). These requirements would not have been viable for the profession over 2 decades ago, shortly after the BACB’s formation.

In its early history, research and practice in behavior analysis was largely regulated by the same mechanisms regulating those of psychology. This is partly because academically oriented behavior analysts were primarily employed in psychology departments; therefore, it seemed logical that they would follow the ethics requirements pertaining to research and practice developed by the American Psychological Association (Johnston et al., 2019). As behavior-analytic service delivery advanced and became more widespread, it began to move towards becoming a distinct profession from clinical psychology. It was at this point that many behavior analysts began to recognize the need for a set of ethics standards that would meet their unique needs (Johnston et al., 2019). The culmination of the establishment of the BACB, the recognition that our profession was different from clinical psychology, and the fact that many behavior analysts were obtaining degrees in disciplines other than psychology (e.g., education) prompted efforts to develop a code of ethics for behavior analysts (G. Green, personal communication, May 12, 2021).

Ethics codes are instrumental in helping members of a profession navigate ethical dilemmas (or avoid them outright), giving the public a mechanism for reporting potentially unethical behavior to certification and regulatory bodies, and providing a basis for those bodies to take disciplinary action (Sellers et al., 2020). As a young profession, applied behavior analysis has had a widely used ethics code for just over 2 decades. By contrast, medicine’s first ethics code was published in 1847 (American Medical Association, n.d.) and psychology’s first ethics code was published in 1953 (Nagy, 2011). Even more junior professions such as occupational therapy, with its first code published in 1977 (American Occupational Therapy Association, n.d.), have had ethics codes for decades longer than applied behavior analysis. These professions and others have periodically revised their ethics codes as their professions and society have evolved. It is important to note that members of these professions have documented the evolution of their ethics codes in journal articles (e.g., Baker & Emanuel, 2000) and white papers (e.g., American Medical Association, n.d.). Such accounts provide important historical information for the scholarly record and can help contextualize current standards with an understanding of what came before. Thus, the purpose of the current article is to present the first history of the BACB’s ethics-related documents and codes.^2^

## Ethics Codes for BCBAs and BCaBAs

### Professional (Disciplinary) Standards (1999)^3^

In 1999, the BACB published and enacted the Professional (Disciplinary) Standards (“Disciplinary Standards”; BACB, 1999). This document included eight standards that described grounds upon which the BACB could issue sanctions against a BACB certificant or applicant. Sanctions included, but were not limited to, denials of initial certification, renewal, or recertification; revocation; suspension; or other limitations on certification. The language in the Disciplinary Standards document focused on describing certificant misconduct related to interactions with the BACB or other entities (e.g., funding agencies, governmental bodies), certain criminal convictions, or the ability to competently practice.

The first standard in the Disciplinary Standards document allowed the BACB to sanction an individual if it was discovered that they were ineligible for certification. For example, the BACB could sanction an individual if it was discovered that a certification was based on false or forged information submitted in their application, regardless of when the ineligibility was discovered. The second disciplinary standard established the ability to sanction for a violation of any BACB rule or procedure or if a certificant failed to update previously submitted information within 30 days (including failing to report certain critical information to the BACB). The third and fourth standards focused on prohibiting the unauthorized possession, use, or distribution of the BACB’s intellectual property (e.g., logos, documents, examination materials); misrepresenting one’s certification status; and engaging in misconduct related to the examination process or materials. The fourth standard added the possible consequence of delaying, canceling, or refusing to release examination results following the detection of examination irregularities. The fifth standard prohibited obtaining or attempting to obtain certification or recertification through false, fraudulent, or misleading interactions with the BACB. Standard six allowed sanctions for gross or repeated negligence, incompetence, or malpractice related to the practice of applied behavior analysis. The seventh disciplinary standard provided a basis for sanction based on certain disciplinary actions (e.g., practice restriction, sanction, revocation, suspension) by a health-care organization, professional organization, or other private or governmental body, if those actions related to the practice of applied behavior analysis, public health or safety, or behavior analysis certification. The eighth and final standard allowed for action based on a felony or misdemeanor conviction directly relating to practice and/or public health and safety.

The Disciplinary Standards document was not an ethics code, but rather a contractual agreement between the BACB and its certificants, who consented to being bound by the Disciplinary Standards when they applied for certification. The Disciplinary Standards represented a modest initial step in helping to protect the integrity of the nascent BACB certification programs and in addressing certificant misconduct that had been firmly established by other organizations.

### Guidelines for Responsible Conduct for Behavior Analysts (2001)

In 1998, the Executive Council of the Association for Behavior Analysis (ABA; now the Association for Behavior Analysis International [ABAI]) charged its Professional Standards Committee with drafting a code of ethics for behavior analysts. This initiative began under the leadership of the committee’s chair, Dr. Gina Green, and was later transferred to the subsequent chair, Dr. John Jacobson (G. Green, personal communication, May 12, 2021). To develop the new code, Dr. Jacobson reviewed the ethics codes of nine professional organizations^4^ in a variety of disciplines (e.g., psychology, social work, school psychology; Johnston et al., 2019). He then conducted two surveys of senior behavior analysts to solicit feedback on a preliminary ethics-code document (Johnston et al., 2017, 2019). Between late 1999 and early 2000, Dr. Jacobson presented a draft of the ethics code to the ABA Executive Council. The ABA Executive Council ultimately voted not to adopt the code and shortly thereafter released an official statement to its members stating that the organization would adopt the American Psychological Association’s code of ethics for its members (G. Green, personal communication, May 12, 2021). Still convinced that there was a need for a code of ethics for behavior analysts given the unique aspects of behavior-analytic practice that differ from psychology, Dr. Shook, who was a member of the ABA Executive Council at the time as well as the BACB’s founder, obtained permission from the ABA Executive Council to present the draft ethics code to the BACB Board of Directors for their consideration (G. Green, personal communication, May 12, 2021). The ethics code was adopted for the BACB and in May 2001, the BACB published and enacted the Guidelines for Responsible Conduct for Behavior Analysts (“Conduct Guidelines”; BACB, 2001)—the organization’s first true ethics code.

The purpose of the Conduct Guidelines (2001) document was to provide ethics-related *guidance* to BCBAs and BCaBAs. Due to the nascence and limited resources of the BACB, which had no salaried full-time staff at that time, the Conduct Guidelines (2001) document was not an enforceable ethics code. However, it operated alongside the Disciplinary Standards (1999) document, which remained the sole mechanism for addressing certificant misconduct (albeit under admittedly limited circumstances). The Conduct Guidelines (2001) document comprised 104 guidelines organized in 10 sections, which covered a variety of topics that pertained to the behavior analyst’s responsibilities to clients, colleagues, and the profession of applied behavior analysis when providing services and conducting research (see Table 1).

**Table 1:** Structural comparison of the BACB’s five ethics codes for BCBAs and BCaBAs from 2001 to 2022

Title	Guidelines for Responsible Conduct for Behavior Analysts	Guidelines for Responsible Conduct for Behavior Analysts	Guidelines for Responsible Conduct for Behavior Analysts	Professional and Ethical Compliance Code for Behavior Analysts	Ethics Code for Behavior Analysts
Enactment Year	2001	2004	2010	2016	2022
Full Enforceability of Code	No	No	No	Yes	Yes
Articulation of Underlying Principles	No	No	No	No	Yes
# of Sections	10	10	10	10	6
# of Elements	104	99	98	71	85
Section Titles	• 1.0 - Responsible Conduct of a Behavior Analyst • 2.0 - The Behavior Analyst's Responsibility to Clients • 3.0 - The Behavior Analyst's Pre-Intervention Behavior • 4.0 - The Behavior Analyst and the Individual Behavior Change Program • 5.0 - The Behavior Analyst as Teacher and/or Supervisor • 6.0 - The Behavior Analyst and the Workplace • 7.0 - The Behavior Analyst and Research • 8.0 - The Behavior Analyst's Ethical Responsibility to the Field of Behavior Analysis • 9.0 - The Behavior Analyst's Ethical Responsibility to Colleagues • 10.0 - The Behavior Analyst's Ethical Responsibility to Society	• 1.0 - Responsible Conduct of a Behavior Analyst • 2.0 - The Behavior Analyst's Responsibility to Clients • 3.0 - Assessing Behavior • 4.0 - The Behavior Analyst and the Individual Behavior Change Program • 5.0 - The Behavior Analyst as Teacher and/or Supervisor • 6.0 - The Behavior Analyst and the Workplace • 7.0 - The Behavior Analyst and Research • 8.0 - The Behavior Analyst's Ethical Responsibility to the Field of Behavior Analysis • 9.0 - The Behavior Analyst's Responsibility to Colleagues • 10.0 - The Behavior Analyst's Ethical Responsibility to Society	• 1.0 - Responsible Conduct of a Behavior Analyst • 2.0 - The Behavior Analyst's Responsibility to Clients • 3.0 - Assessing Behavior • 4.0 - The Behavior Analyst and the Individual Behavior Change Program • 5.0 - The Behavior Analyst as Teacher and/or Supervisor • 6.0 - The Behavior Analyst and the Workplace • 7.0 - The Behavior Analyst's Ethical Responsibility to the Field of Behavior Analysis • 8.0 - The Behavior Analyst's Responsibility to Colleagues • 9.0 - The Behavior Analyst's Ethical Responsibility to Society • 10.0 - The Behavior Analyst and Research	• 1.0 - Responsible Conduct of Behavior Analysts • 2.0 - Behavior Analysts’ Responsibility to Clients • 3.0 - Assessing Behavior • 4.0 - Behavior Analysts and the Behavior-Change Program • 5.0 - Behavior Analysts as Supervisors • 6.0 - Behavior Analysts’ Ethical Responsibility to the Profession • 7.0 - Behavior Analysts’ Ethical Responsibility to Colleagues • 8.0 - Public Statements • 9.0 - Behavior Analysts and Research • 10.0 - Behavior Analysts’ Ethical Responsibility to the BACB	• 1 - Responsibility as a Professional • 2 - Responsibility in Practice • 3 - Responsibility to Clients and Stakeholders • 4 - Responsibility to Supervisees and Trainees • 5 - Responsibility in Public Statements • 6 - Responsibility in Research

The first section, Responsible Conduct of a Behavior Analyst, focused on general conduct (e.g., integrity, reliance on science, competence) and ethics-related issues related to professional and personal relationships (e.g., harassment and discrimination, dual and exploitative relationships). Section 2, The Behavior Analyst’s Responsibility to Clients, described the expectations related to business aspects of service delivery such as fees and financial arrangements, confidentiality, record keeping, and terminating services. Sections 3 and 4 (The Behavior Analyst’s Pre-Intervention Behavior and The Behavior Analyst and the Individual Behavior Change Plan, respectively) pertained to direct service delivery. These sections included guidelines related to conducting behavioral assessments, providing services within one’s scope of competence, and designing and implementing behavior-change interventions. The fifth section, The Behavior Analyst as a Teacher and/or Supervisor, included guidance related to teaching (e.g., providing clear descriptions, expectations, and evaluation criteria) and supervisory activities (e.g., feedback, positive reinforcement, publication credit). Section 6, The Behavior Analyst and the Workplace, focused on the behavior analyst’s responsibilities towards their organization and employees (e.g., adhering to commitments, developing interventions that benefit employees and enhance health and wellness). The seventh section, The Behavior Analyst and Research, was the longest section, and included guidance related to confidentiality, deception, participant recruitment, and research with animals. The remaining three sections—The Behavior Analyst’s Ethical Responsibility to the Field of Behavior Analysis, The Behavior Analyst’s Ethical Responsibility to Colleagues, and The Behavior Analyst’s Ethical Responsibility to Society—outlined the behavior analyst’s responsibilities with respect to upholding and advancing the values and principles of behavior analysis, dissemination of behavior analysis, honesty in the representation and publication of data, statements related to behavior analysis as a discipline, and the individual behavior analyst’s work, and testimonials and in-person solicitation.

### Guidelines for Responsible Conduct for Behavior Analysts (2004)

In 2004, the BACB published and enacted the first revision of the Conduct Guidelines (2001) document (BACB, 2004). Dr. Jon Bailey served as chair of the revision workgroup, which included multiple SMEs with expertise in applied behavior analysis and in teaching applied ethics. The revised version of the Conduct Guidelines (2001) document was not substantively different than the prior version and can be considered a technical revision. In the original Conduct Guidelines (2001) document, the guidelines progressed within a numbered section by increasing the middle and last numbers separated by decimals (e.g., 7.19, 7.19.1, 7.19.2), and titles were not consistently used for each enumerated guideline. In the 2004 revision, there were 99 guidelines organized in 10 sections, but titles were developed for each guideline and in each section, the standards were enumerated using an X.0X format (see Table 1). In addition, guidelines with multiple subsections were organized using lowercase letters in parentheses (e.g., 1.08[a]). The 2004 revision also added a summary statement at the beginning of each section following the primary section number and title. For example, the first section was titled 1.0 Responsible Conduct of a Behavior Analyst and was followed by the statement “The behavior analyst maintains the high standards of the professional behavior of the professional organization.”

Whereas most of the guideline language in the 2004 version remained the same, one addition was the summary statement following section 2.0 The Behavior Analyst’s Responsibility to Clients that read: “The behavior analyst has a responsibility to operate in the best interest of clients.” This text better articulated “The Behavior Analyst’s Responsibility to Clients” than the 2001 version text which simply defined “client.” Another notable change was that standard 5.9 Principal Authorship and Other Publication Credits from the 2001 version was moved into section 7.0 The Behavior Analyst and Research. Finally, standards 7.19.1 through 7.19.8, specific to animal research, were removed entirely from Section 7, because behavior analysts conducting animal research generally had other institutional and ethical systems governing such activity (e.g., Institutional Animal Care and Use Committees).

### Professional Disciplinary and Ethical Standards (2010)

Efforts to disseminate the revised Conduct Guidelines (2004) document included delivering presentations and workshops at behavior analysis conferences where Dr. Shook and members of the BACB Board of Directors summarized the document’s content and solicited feedback from attendees (J. S. Bailey, personal communication, May 26, 2021). Through these activities the BACB identified the need to increase its ability to take action—through the Disciplinary Standards (1999) document—against an individual’s certification when they had engaged in professional misconduct. Until this point, the BACB could typically only take action against an individual’s certification under the Disciplinary Standards (1999) in instances of gross or repeated negligence or malpractice where there was documentation that an entity (e.g., employer, governmental agency, court of law) charged with overseeing the case, client, or certificant conducted a review and took prior disciplinary action.

To broaden the BACB’s ability to address professional misconduct by its certificants, the BACB Task Force on Professional Disciplinary Standards was created in 2007. Although there were no specific instances of unethical behavior that led to the task force, by 2007 the number of BACB certificants had increased to just over 5,000 with a modest increasing trend. Thus, it was prudent to expand and develop systems in preparation for future needs. The task force was chaired by Dr. Jim Johnston and its SMEs (e.g., practitioners, faculty members) were charged with reviewing the Disciplinary Standards (1999) document and making revision recommendations. Those recommendations were reviewed and adopted by the BACB Board of Directors in 2008 and the revised document—Professional Disciplinary and Ethical Standards (BACB, 2010a) —was enacted on January 1, 2010. The changes included adding the word “misconduct” into disciplinary standard 6 to explicitly link that standard to the Conduct Guidelines document and providing numerous examples of misconduct (BACB, 2008). These included deviating from customary practices resulting in serious risk to consumers, consumer abandonment without proper notice and transition, inappropriate record keeping or falsifying data, engaging in fraud, and disclosing unauthorized confidential consumer information. The change to disciplinary standard 6 was critical, as the added text gave the BACB the ability to independently accept cases of gross or repeated misconduct and determine whether the complaint should proceed to a Review Committee for possible action. Another notable change to the Disciplinary Standards document was the addition of standard 9, which provided a basis for action in instances when certificants failed to receive or provide supervision in accordance with BACB supervision standards. Finally, adding the word “ethical” to the document title reflected the expanded scope of activity that was actionable under standard 6, which explicitly linked the Conduct Guidelines (2010) document (the ethics code) to the Disciplinary Standards Document (BACB, 2010b).

### Guidelines for Responsible Conduct for Behavior Analysts (2010)

During the BACB Task Force on Professional Disciplinary Standards meeting, SMEs conducted a thorough review of the Conduct Guidelines (2004) document to guide their review of the Disciplinary Standards (1999) document. During this review, it was apparent that the Conduct Guidelines (2004) document required another technical revision for concision and clarity. In addition, the recently developed *Fourth Edition Task List* (BACB, 2012) for the BCBA and BCaBA examinations, along with corresponding changes in educational requirements, made another review of the Conduct Guidelines (2004) document prudent. Dr. Bailey was subsequently tasked with undertaking another SME-based review of the document for possible revision. This review was conducted virtually involved practicing and academic behavior analysts.

The revised version of the Conduct Guidelines document (BACB, 2010c) included numerous text revisions, title revisions, a reorganization of sections, and new guidelines. The 98 guidelines remained organized in 10 sections (see Table 1). The new guideline 1.03 Professional Development incorporated the text from the former 1.02 Competence and Professional Development. Subguideline (a) requiring behavior analysts to be truthful and honest and follow through on commitments was added to 1.04 Integrity. The title for 1.06 was expanded to include conflicts of interest. Guideline 3.04 Accepting Clients from the 2004 version was moved into section 2.0, The Behavior Analyst’s Responsibility to Clients, as guideline 2.02. Other changes to section 2.0 include adding sub-guideline (e) to 2.06 Rights and Prerogatives of Clients, subguideline (d) to 2.10 Treatment Efficacy, and subguideline (c) to 2.13 Fees, Financial Arrangements and Terms of Consultation. In section 3.0 Assessing Behavior, the primary changes included additions to the language in 3.01 Behavioral Assessment Approval from old 3.06(b) and in 3.05 Describing Program Objectives around conducting a risk-benefit analysis. Several guidelines from former section 3.0 (3.01, 3.03, and 3.08) were moved into section 4.0 The Behavior Analyst and the Individual Behavior Change Program. The language in the guidelines in sections 4.0, 5.0 The Behavior Analyst as Teacher and/or Supervisor, and 6.0 The Behavior Analyst and the Workplace remained relatively unchanged. The last four sections were reordered such that The Behavior Analyst’s Ethical Responsibility to the Field of Behavior Analysis and The Behavior Analyst’s Ethical Responsibility to Colleagues appeared before the section covering The Behavior Analyst’s Ethical Responsibility to Society and The Behavior Analyst and Research. The remaining four sections were reordered, and some guidelines were reassigned to different sections. The most notable changes include added language to 8.01 Ethical Violations by Behavioral and Non-behavioral Colleagues for what to do if a resolution to an apparent ethics violation is not obtained, and the removal of 9.06(b) related to use of media in service delivery or teaching.

### Professional and Ethical Compliance Code for Behavior Analysts (2016)

Although the 2010 version of the Conduct Guidelines document had only been in effect for three years, in late 2013 the BACB Board of Directors authorized another SME-based review of it and the Disciplinary Standards (2010) for possible revision. The profession had continued to grow in recent years and its need for a fully enforceable ethics code was increasingly apparent. By late 2013, the number of BACB certificants was close to 15,000 with an accelerating upward trend. The growth in the number of certificants suggested a sharp growth in service activity. The BACB now had sufficient staff and resources such that a fully enforceable ethics code was now within the realm of possibility. An enforceable ethics code and a more robust system for handling ethics complaints would likely soon be needed. In advance of the review, feedback on the 2010 Conduct Guidelines document was gathered from multiple sources, including ABAI’s Ethics and Behavior Analysis Special Interest Group and behavior analyst licensure board representatives (BACB, 2014a).

A workgroup chaired by Dr. Bailey, met for three days in Chicago, IL on May 21–23, 2014, to evaluate the BACB’s ethics standards (BACB, 2014a). The workgroup included consumer representatives and behavior analysts who represented a range of professional roles (e.g., practitioner, faculty member, licensure board representative, disciplinary review committee member) from eight U.S. states and two countries. Each ethics standard was reviewed and revised by the committee using a consensus approach. The workgroup also provided guidance for a system for managing disciplinary complaints that expanded the BACB’s current system. The expanded system included two committees for reviewing complaints based on severity of the allegations, an online complaint-submission system, and public reporting of certain sanctions (see BACB, 2014a for additional detail). The BACB Board of Directors approved the workgroup recommendations in August 2014.

The new ethics code—the Professional and Ethical Compliance Code for Behavior Analysts (PECC) —was published by the BACB in September 2014 and became effective on January 1, 2016 (BACB, 2014b). The PECC included 71 elements (and 169 individual standards) organized in 10 sections (see Table 1). The PECC also included a table of contents and glossary, a first for the organization’s ethics-based documents. Numerous edits were made to the prior guidelines that appeared in the PECC. For example, element 1.06(d) was added under Multiple Relationships and Conflict of Interest to clarify that behavior analysts do not accept or give gifts to clients. Element 1.07(c) was added to Exploitative Relationships, specifying that behavior analysts do not engage in sexual relationships with clients, students, or supervisees for at least 2 years after the professional relationship has formally ended. Section 5.0 removed references to teachers and now focused exclusively on supervisory relationships, as university and college faculty have their own institutional standards. Supervisory volume was addressed with the addition of 5.02, and 5.07 was added to directly address the evaluation of supervision activities. Section 6.0 The Behavior Analyst and the Workplace was removed from the code and instead was addressed under sections 2.12 and 10.02(a). Element 8.02(b) under Avoiding False and Deceptive Statements was substantially revised to better address individuals who provide a combination of behavior-analytic and nonbehavior-analytic interventions. Element 8.04 Media Presentations and Media-Based Services was updated to highlight the importance of the code in these activities and to provide some clarification with issues related to social and other media. Element 8.05 Testimonials and Advertising now included the requirement that all testimonials from former clients be labeled as solicited or unsolicited, and provided additional guidance on the practice. Section 9.0 Behavior Analysts and Research was revised to highlight a behavior analyst’s responsibility in research, but eliminated the specific research requirements routinely imposed by research-oversight bodies, such as institutional review boards. Section 10.0 Behavior Analysts’ Ethical Responsibility to the BACB covered many of the Professional Disciplinary and Ethical Standards (2010), as well as other BACB requirements.

### Ethics Code for Behavior Analysts (2022)

In 2018, the BACB began a lengthy process for revising the PECC with the goal of making it more efficient and readable, and to address the rapid pace of changes in the profession (BACB, 2020a). As the first step in the process, feedback about the PECC that had been submitted to the BACB was compiled and reviewed. A review of ethics codes^5^ of other helping professions was also conducted to evaluate their content and structure. This review helped inform the new code’s preamble, guiding principles, and revisions to ethics standards about gifts, multiple relationships, service transition, supervision, self-reporting, and client testimonials. A survey was distributed to all BCBAs and BCaBAs to solicit feedback on the PECC, resulting in 4,728 responses (approximately 11% of all BCBAs and BCaBAs at that time). Another survey was also distributed to behavior analysts with expertise in ethics and representatives from behavior analyst licensure boards. The information from the surveys was reviewed, summarized, and used in the revision process. Multiple SME groups met virtually to review the survey data, discuss ethics issues, and revise code standards. A total of 26 SMEs were involved in the revision representing a range of professional experience and practice areas (e.g., teaching ethics, engaging in ethics-related scholarly work, supervising, holding dual certification/licensure). A final workgroup of seven SMEs completed one last review of the new code and provided additional feedback. In July 2020, the BACB Board of Directors reviewed and approved the new code, the Ethics Code for Behavior Analysts (code), which was published in December 2020 and enacted on January 1, 2022 (BACB, 2020b).

The new code included an introduction section describing its scope and the BACB’s jurisdiction, the code’s four main underlying principles, how the code should be applied, considerations for ethical decision making, and code-enforcement procedures. The glossary was moved to the beginning of the document, between the Introduction and Standard Sections, to increase the likelihood of its use. The code consists of 85 standards organized in six sections (see Table 1).

Many changes occurred during the revision process. Several notable changes were made to Section 1. Standard 1.07 Cultural Responsiveness and Diversity was added to clarify that behavior analysts are required to engage in professional development activities related to cultural responsiveness and diversity and evaluate their own biases and abilities when serving those with diverse needs and backgrounds. Standard 1.08 Nondiscrimination was updated from its version in the PECC to focus on requiring equitable and inclusive behavior and to include additional protected groups and statuses. Standard 1.10 was revised to focus on personal biases and challenges, specifically being aware of and addressing them. The standard related to gift giving (1.12 Giving and Receiving Gifts) became a stand-alone standard and was revised to indicate that gift exchanges are allowed under $10 (or the equivalent purchasing power in another country’s currency). Standard 1.14 Romantic and Sexual Relationships was separated and revised with additional time frames, contexts, and considerations. In Section 2, Responsibility in Practice, 2.08 Communicating about Services was updated to require behavior analysts to ensure that clients, stakeholders, supervisees, trainees, and research participants comprehend the information communicated to them. The standard related to consent (2.11 Obtaining Informed Consent) now clarified the conditions under which consent and assent must be obtained.

In Section 3, Responsibility to Client and Stakeholders, standards 3.07, 3.08, and 3.09 were revised to clarify the obligations of behavior analysts when providing services through a third party, including the need to document all actions taken to address any conflicts between the best interests of the client and the directions of the third party. In addition, standards 3.14, 3.15, and 3.16 were revised to clarify the expectations of behavior analysts when managing service interruptions, transitions, and discontinuation. Section 4, Responsibility to Supervisees and Trainees had multiple changes, including 4.03 Supervisor Volume, which includes more considerations for supervisory capacity and 4.04 Accountability in Supervision, which set the expectation that behavior analysts are responsible for their supervisees’ and trainees’ professional activities. Standard 4.07, Incorporating and Addressing Diversity was a new standard addressing the inclusion of diversity in training and supervision activities. Standards 4.11 and 4.12 are new, but an extension of the related standards in Section 3. In Section 5, Responsibility to Clients, 5.03 Public Statements by Behavior Analysts was updated to address providing client-specific advice in public forums. Standards 5.07, 5.08, and 5.09 were revised to guide behavior analysts through the requirements for using client testimonials, and 5.10, Social Media Channels and Websites, was revised with a distinction between professional and personal social media channels and websites. Finally, Section 6, Responsibility in Research, included a new standard, 6.03, addressing research conducted during service delivery and revisions to 6.04 to include expectations for using existing client data.

## Ethics Codes for RBTs

The BACB announced the new RBT certification in 2013 (BACB, 2013). This new paraprofessional certification was designed for behavior technicians, the individuals who often implement behavior plans under the supervision of a BCBA or BCaBA. The SMEs who developed the initial RBT requirements did not recommend a new ethics code for RBTs, but instead, identified 29 guidelines of the 2010 Conduct Guidelines document by which RBTs must abide (BACB, 2013). These guidelines constituted the Conduct Guidelines that were most relevant to the work of behavior technicians. The following year during the meeting to develop the PECC, the SMEs used a similar approach to identify the standards that would apply to RBTs. The SMEs reviewed each PECC standard to identify whether it was appropriate for RBT-level work activities or whether it would hold an individual to a level of duty that was not commensurate with the scope of their work activities (J. S. Bailey, personal communication, May 26, 2021). The group eventually identified 33 elements across all sections that were relevant to RBTs, except Section 5.0 Behavior Analysts as Supervisors. The RBT-relevant elements were indicated with a superscript “RBT” in red next to the standard title in the Table of Contents and throughout the body of the document (BACB, 2014b).

### RBT Ethics Code (2019)

In 2017, an SME workgroup convened to review RBT certification requirements recommended that an ethics code be developed specifically for RBTs to better highlight their ethical obligations and to present them in a more understandable manner (BACB, 2018a). The ethics standards designated for RBTs in the PECC were reevaluated for their current appropriateness for RBTs, rewritten at the 12th-grade level, and organized into three sections: Responsible Conduct, Responsibility to Clients, and Competence and Service Delivery. The 31-standard RBT Ethics Code (BACB, 2018b) was published in December 2018 and was enacted on January 1, 2019. Because the RBT Ethics Code did not include any new ethical obligations for RBTs, it was able to be enacted immediately.

### RBT Ethics Code (2.0; 2022)

Because the RBT Ethics Code was based on standards in the PECC that would soon be replaced by the Ethics Code for Behavior Analysts, the BACB implemented a revision project for the RBT Ethics Code in October 2020. There were two primary goals for that project: (1) to bring the RBT ethics standards in line with those in the Ethics Code for Behavior Analysts; and (2) to revise the content of the standards such that they were relevant to the typical work activities of RBTs. That revision project included (1) reviewing archived feedback; (2) reviewing four relevant codes of ethics from other organizations^6^ that certify technician- or assistant-level workers; (3) surveying active RBTs and all BCaBA/BCBA certificants whose BACB records indicated that they served as an RBT Supervisor or RBT Requirements Coordinator; and (4) holding several SME workgroups. The revised RBT Ethics Code (2.0) was presented to and approved by the BACB Board of Directors in May 2021 for implementation on January 1, 2022 to parallel the enactment of the Ethics Code for Behavior Analysts. The RBT Ethics Code (2.0; BACB, 2021a) was published in July 2021 (BACB, 2021b).

The RBT Ethics Code (2.0) is comprised of the following main sections: Introduction, Ethics Standards, and Glossary. The Introduction is modeled on the introduction section in the Ethics Code for Behavior Analysts but was edited for relevance to RBTs. The introduction section includes brief descriptions of the scope of the RBT Ethics Code (2.0), the four core principles that serve as its foundation, and how to apply the new code. This section also clearly indicates that RBTs should rely on their BACB-required supervisor when applying the new code. The document’s 29 standards are organized in three sections: (1) General Responsibilities; (2) Responsibilities in Providing Behavior-Technician Services; and (3) Responsibilities to the BACB and BACB-required Supervisor. The Glossary, which did not appear in the previous version of the RBT Ethics Code, contains 10 terms (9 that also appear in the Ethics Code for Behavior Analysts).

## Conclusion

As of this writing, the BACB has been in existence for over 25 years. During that time, the applied behavior analysis profession has changed dramatically. There are now over 200,000 BACB certificants (BACB, n.d.-a), 36 U.S. states have enacted licensure for behavior analysts since 2009 (BACB, n.d.-b), and demand for behavior-analytic practitioners has increased every year for over a decade (BACB, n.d.-c). Indeed, just about every aspect of our professional infrastructure has changed in recent years. During this time, the BACB has steadily revised its practices and standards as the profession has changed and the BACB’s capabilities have increased.

**Fig. 1:**
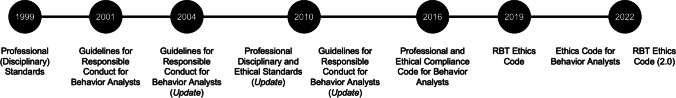
Historical timeline of BACB ethics-related documents and codes and their enactment dates

To date, the BACB has developed and published nine distinct ethics-related documents for its certificants: two sets of disciplinary standards for BCBAs and BCaBAs, five ethics codes for BCBAs and BCaBAs (see Table 1 for a comparison), and two ethics codes for RBTs (see Fig. 1). This was a substantial amount of activity that was stimulated by a rapidly changing professional landscape. These changes included widespread availability of funding for behavior-analytic services, which led to the development of hundreds of new service organizations and hundreds of new university training programs, and ultimately over 200,000 BACB certificants. In addition, during this time the BACB’s operational capacity grew from one unpaid employee in 1998 (Dr. Shook) to over 100 full-time employees at the end of 2022. This increased operational capacity meant that the BACB could initiate and refine its ethics infrastructure to meet the needs of a rapidly growing profession. The BACB’s evolving ethics-related documents have been instrumental in establishing ethics standards, guiding ethical decision making, and serving as a basis for disciplinary action. In addition to the BACB’s codes serving as the basis for the BACB’s code-enforcement activities, the current BACB codes are used by almost half of all U.S. licensure programs as their enforceable ethics standards.

In addition to the promulgation of written ethics standards, the BACB has also contributed to the profession’s education in ethics. In 2003, the BACB’s coursework and training requirements began including explicit instruction in ethics. These requirements began at a modest state and have been increased over time. Today, BCBA and BCaBA coursework requirements include 45 and 30 hr of ethics instruction, respectively, and 3 of the 40 RBT initial training hr must be devoted to ethics. In addition, the BACB instituted an ethics subcategory in its continuing education requirements beginning in 2008. Today, BCBAs and BCaBAs are required to complete 4 hr of continuing education in ethics every 2 years.

Through its well-refined ethics codes, required coursework and training in ethics, and required continuing education in ethics, the BACB has helped shape the ethical foundation of our profession. As our professional infrastructure and society continue to change, there will undoubtedly be further refinements in these areas, although perhaps less frequently than in the past 25 years.

## Data Availability

Data sharing is not applicable to this article as no data sets were generated or analyzed during the current study.

## Footnotes

1 We refer the reader to Iwata et al. (2011) for additional information about Dr. Shook’s involvement in the Florida certification program and the BACB.

2 As is to be expected from such an effort, descriptions of the organization’s early activities include fewer details than descriptions of later activities due to lack of comprehensive documentation from the organization’s first decade of operation.

3 The years that accompany document names in section headings represent their enactment, not their original publication.

4 American Anthropological Association, American Educational Research Association, American Psychological Association, American Sociological Association, California Association for Behavior Analysis, Florida Association for Behavior Analysis, National Association of Social Workers, National Association of School Psychologists, and Texas Association for Behavior Analysis.

5 American Speech-Language-Hearing Association; California Association of Marriage and Family Therapists; Canadian Psychological Association; National Association of Social Workers; and National Board for Certification in Occupational Therapy.

6 American Speech-Language-Hearing Association; Behavioral Intervention Certification Council; National Board for Certification in Occupational Therapy; and Qualified Applied Behavior Analysis Credentialing Board.

## References

[CR1] American Medical Association (n.d.). *History of the code*. https://www.ama-assn.org/sites/ama-assn.org/files/corp/media-browser/public/ethics/ama-code-ethics-history.pdf. Accessed 27 Apr 2023

[CR2] American Occupational Therapy Association. (n.d.). *Important events.* http://www.otcentennial.org/100-events/1977. Accessed 27 Apr 2023

[CR3] Baker, R., & Emanuel, L. (2000). The efficacy of professional ethics: The AMA code of ethics in historical and current perspective. *The Hastings Center Report, 30*(4), S13–S17. 10.2307/3527657 10971897

[CR4] Behavior Analyst Certification Board. (n.d.-a). *BACB certificant data*. https://www.bacb.com/bacb-certificant-data/. Accessed 27 Apr 2023

[CR5] Behavior Analyst Certification Board. (n.d.-b). *U.S. licensure of behavior analysts.* https://www.bacb.com/u-s-licensure-of-behavior-analysts/. Accessed 27 Apr 2023

[CR6] Behavior Analyst Certification Board. (n.d.-c). *U.S. employment demand for behavior analysts:2010–2022.* https://www.bacb.com/wp-content/us_employmentdemand_ba/. Accessed 27 Apr 2023

[CR7] Behavior Analyst Certification Board. (1999). *Professional (disciplinary) standards*. https://www.bacb.com/wp-content/uploads/2020/09/1999-Disciplinary-Standard.pdf. Accessed 27 Apr 2023

[CR8] Behavior Analyst Certification Board. (2001). *Guidelines for responsible conduct for behavior analysts*. https://www.bacb.com/wp-content/uploads/2020/09/2001-Conduct-Guidelines.pdf. Accessed 27 Apr 2023

[CR9] Behavior Analyst Certification Board. (2004). *Guidelines for responsible conduct for behavior analysts*. https://www.bacb.com/wp-content/uploads/2020/09/2004-Conduct-Guidelines.pdf. Accessed 27 Apr 2023

[CR10] Behavior Analyst Certification Board. (2008). Improvements and clarification of the disciplinary standards. *BACB Newsletter*. https://www.bacb.com/wp-content/uploads/2020/05/BACB_Newsletter_9_08.pdf. Accessed 27 Apr 2023

[CR11] Behavior Analyst Certification Board. (2010a). *Professional disciplinary and ethical standards*. https://www.bacb.com/wp-content/uploads/2020/09/2010-Disciplinary-Standards_.pdf. Accessed 27 Apr 2023

[CR12] Behavior Analyst Certification Board. (2010b) The BACB professional disciplinary and ethical standards . . . and process. . https://www.bacb.com/wp-content/uploads/2020/05/BACB_Newsletter_05_2010.pdf. Accessed 27 Apr 2023

[CR13] Behavior Analyst Certification Board. (2010c). *Guidelines for responsible conduct for behavior analysts*. https://www.bacb.com/wp-content/uploads/2020/09/2010-Conduct-Guidelines.pdf. Accessed 27 Apr 2023

[CR14] Behavior Analyst Certification Board. (2012). *Fourth edition task list*. Tallahassee, FL: Author

[CR15] Behavior Analyst Certification Board. (2013). Special edition on the RBT credential*. BACB Newsletter.* https://www.bacb.com/wp-content/uploads/2020/05/BACB_Newsletter_12-13.pdf. Accessed 27 Apr 2023

[CR16] Behavior Analyst Certification Board. (2014a). Special edition on ethics*. BACB Newsletter.* https://www.bacb.com/wp-content/uploads/2020/05/BACB_Newsletter_09-14.pdf. Accessed 27 Apr 2023

[CR17] Behavior Analyst Certification Board. (2014b). *Professional and ethical compliance code for behavior analysts.* https://www.bacb.com/wp-content/uploads/2022/05/BACB-Compliance-Code-10-8-15watermark.pdf. Accessed 27 Apr 2023

[CR18] Behavior Analyst Certification Board. (2018a). Special edition: RBT certification changes and process improvements. *BACB Newsletter*. https://www.bacb.com/wp-content/uploads/2020/05/BACB_December2018_Newsletter-200828.pdf. Accessed 27 Apr 2023

[CR19] Behavior Analyst Certification Board. (2018b). *RBT ethics code*. https://www.bacb.com/wp-content/uploads/2020/05/RBT-Ethics-Code_190227-historical.pdf. Accessed 27 Apr 2023

[CR20] Behavior Analyst Certification Board. (2020a). Introducing the new ethics code for behavior analysts. *BACB Newsletter*. https://www.bacb.com/wp-content/uploads/2020/12/BACB_December2020_Newsletter-210624.pdf. Accessed 27 Apr 2023

[CR21] Behavior Analyst Certification Board. (2020b). *Ethics code for behavior analysts*. https://bacb.com/wp-content/ethics-code-for-behavior-analysts. Accessed 27 Apr 2023

[CR22] Behavior Analyst Certification Board. (2021a). *RBT ethics code (2.0)*. https://www.bacb.com/wp-content/uploads/2022/01/RBT-Ethics-Code-220316-2.pdf. Accessed 27 Apr 2023

[CR23] Behavior Analyst Certification Board. (2021b). *BACB Newsletter*. https://www.bacb.com/wp-content/uploads/2022/01/BACB_July2021_Newsletter-220131.pdf. Accessed 27 Apr 2023

[CR24] Iwata, B. A., Sundberg, M. L., & Carr, J. E. (2011). Gerald L. “Jerry” Shook: Visionary for the profession of behavior analysis. *Behavior Analysis in Practice, 4*(2), 61–63. 10.1007/BF03391785 22649580 10.1007/BF03391785 PMC3357102

[CR25] Johnston, J. M., Carr, J. E., & Mellichamp, F. H. (2017). A history of the professional credentialing of applied behavior analysts. *The Behavior Analyst, 40*(2), 523–538. 10.1007/s40614-017-0106-9 31976964 10.1007/s40614-017-0106-9 PMC6701231

[CR26] Johnston, J. M., Mellichamp, F. H., Shook, G. L., & Carr, J. E. (2014). Determining BACB examination content and standards. *Behavior Analysis in Practice, 7*(1), 3–9. 10.1007/s40617-014-0003-6 27057483 10.1007/s40617-014-0003-6 PMC4711728

[CR27] Johnston, J. M., Pennypacker, H. S., & Green, G. (2019). *Strategies and tactics of behavioral research and practice* (4^th^ ed.) . 10.4324/9781315537085

[CR28] Johnston, J. M., & Shook, G. L. (2001). A national certification program for behavior analysts. *Behavioral Interventions, 16*(2), 77–85. 10.1002/bin.81

[CR29] Nagy, T. F. (2011). A brief history and overview of the APA ethics code. In T. F. Nagy (Ed.), *Essential ethics for psychologists: A primer for understanding and mastering core issues* (pp. 29–48). American Psychological Association. 10.1037/12345-002

[CR30] Sellers, T. P., Carr, J. E., & Nosik, M. R. (2020). On the BACB’s ethics requirements: A response to Rosenberg and Schwartz (2019). *Behavior Analysis in Practice, 13*(3), 714–717. 10.1007/s40617-020-00463-6 32953399 10.1007/s40617-020-00463-6 PMC7471222

[CR31] Starin, S., Hemingway, M., & Hartsfield, F. (1993). Credentialing behavior analysts and the Florida behavior analysis certification program. *The Behavior Analyst, 16*(2), 153–166. 10.1007/BF03392620 22478143 10.1007/BF03392620 PMC2733630

